# Outreach by Municipal Public Health Nurses to Vulnerable Pregnant Women During the COVID-19 Pandemic: A Qualitative Study

**DOI:** 10.7759/cureus.78755

**Published:** 2025-02-08

**Authors:** Miyuki Ishii, Junko Honda

**Affiliations:** 1 College of Nursing Art and Science, University of Hyogo, Akashi, JPN; 2 Research Institute of Nursing Care for People and Community, University of Hyogo, Akashi, JPN

**Keywords:** covid-19, maternal health, outreach, public health nursing, vulnerable families

## Abstract

Background: The coronavirus disease 2019 (COVID-19) pandemic posed substantial challenges for public health nurses (PHNs) in delivering outreach services to vulnerable pregnant women and families requiring assistance. Despite restrictive measures, the importance of maintaining face-to-face support and implementing adaptive strategies was evident.

Objectives: This study aimed to identify the key characteristics of outreach practices conducted by PHNs during the pandemic, the challenges they encountered, and the strategies needed to address these issues effectively.

Methods: A qualitative descriptive study was conducted using semi-structured interviews with 11 PHNs from seven Japanese municipalities of varying population sizes. Data were analyzed using thematic coding, focusing on outreach characteristics, challenges, and resource requirements.

Results: The outreach practices included maintaining face-to-face home visits, fostering trust through casual interactions and written correspondence, leveraging collaborative networks, and utilizing digital tools. Major challenges involved balancing infection control with face-to-face support, overcoming the limitations of online consultations, and ensuring service accessibility for families without internet connectivity. Participants advocated for integrated digital tools, flexible communication approaches, and robust data management systems.

Conclusions: Our findings underscore the resilience and adaptability of PHNs in supporting vulnerable families during crises. Addressing these challenges can enhance outreach practices and provide valuable insights for responding to future pandemics or similar emergencies.

## Introduction

The Ministry of Health, Labor, and Welfare [1] issued a notification aimed at preventing child abuse by identifying individuals with concerns related to pregnancy, childbirth, and childcare at an early stage and enhancing parental caregiving capacities through inter-organizational collaboration. In Japan, early interventions targeted at pregnant and postpartum women and their families experiencing psychological, mental, and economic challenges have been expanded. In response, related studies have been conducted, revealing that early interventions for pregnant and postpartum women facing overlapping challenges contribute to the prevention of postpartum depression and child abuse [2-5]. Against this backdrop, legal frameworks, such as the Child Welfare Act, have been revised and strengthened, establishing mechanisms for the early identification and support of pregnant women requiring specific care and families in need. These frameworks have established the foundation for early interventions, which are operationalized through the efforts of municipal public health nurses (PHNs) in providing targeted support to high-risk households.

Traditionally, municipal PHNs have adopted outreaches to provide family and childcare support to high-risk households, including specific pregnant women and families in need. Ongoing support through home visits by PHNs has been shown to facilitate psychological stability in pregnant women, thereby enhancing their capacity to develop essential parenting skills that are indispensable for successful child-rearing. Establishing connections with essential services can mitigate risks such as financial constraints and social isolation, thereby addressing social vulnerabilities and fostering self-reliance. Collectively, these interventions play a pivotal role in supporting the healthy development of children [6-8]. Despite the critical role of PHNs in supporting vulnerable populations during the COVID-19 pandemic, limited studies have examined the specific strategies they employed to address emerging challenges, highlighting the need for further investigation.

In particular, during the coronavirus disease 2019 (COVID-19) pandemic, 42.3% of municipalities reported being unable to conduct home visits for high-risk individuals [9]. This indicates that PHNs encountered substantial difficulties in conducting home visits and executing outreach activities during the pandemic. Nevertheless, the specific realities of these challenges remain unclear.

Therefore, this study aimed to investigate the following dimensions of childcare support for specific pregnant women and families in need during the COVID-19 pandemic: (1) the features of outreach practices performed by municipal PHNs, (2) the challenges they faced, and (3) the resources and support systems required to address these challenges.

## Materials and methods

Operational terms

In this study, specific terms were defined as follows: the COVID-19 pandemic was defined as the period from February 28, 2020, when the Ministry of Health, Labor, and Welfare issued the notification regarding responses to COVID-19 in maternal and child health services, to May 8, 2023, when COVID-19 was reclassified as a Category V infectious disease under the Infectious Diseases Control Act.

Outreach was defined based on a previous study [10] as the proactive efforts by PHNs to engage individuals or families in need of support who have not yet received it. This includes identifying latent needs and delivering preventive support.

Specific pregnant women were defined, according to the Child Welfare Act, as pregnant women experiencing circumstances such as economic hardship, unintended pregnancies, or young age, who require support from the prenatal stage due to anticipated challenges in postnatal caregiving.

Study design

A qualitative descriptive design was adopted, as it is suitable for exploring relatively new research fields and aims to describe phenomena or events in everyday language to enhance understanding [11].

Participants recruitment

Participants were recruited from seven municipalities classified by population size, as this factor was thought to influence the activities and challenges experienced by PHNs during the COVID-19 pandemic. In "larger municipalities," PHNs often manage higher caseloads, work within more complex administrative structures, and have access to more resources, but may face challenges related to bureaucracy and interdepartmental coordination. In contrast, PHNs in "smaller municipalities" typically manage fewer cases, allowing for more personalized care, but may face resource and staffing constraints. To ensure diversity, municipalities were categorized into four groups based on population size: population under 50,000 as small; population between 50,000 and 99,999 as lower middle; population between 100,000 and 499,999 as upper medium; and population of 500,000 or more as large.

Eligible participants were PHNs employed by municipal governments who had experience providing maternal and child health services and assisting specific pregnant women and families in need during the pandemic.

To recruit participants, the researchers sent requests for cooperation and candidate selection guidelines to the heads of maternal and child health departments in each municipality. Depending on the number of PHNs in each municipality, one to three candidates were requested to be selected. The department heads were asked to inform candidates that participation was voluntary. After receiving the contact information forms from the candidates, the researchers contacted them to confirm their willingness to participate. Data collection dates and methods were determined based on the preferences of the 11 participants who consented.

Data collection

Semi-structured interviews were conducted between July and December 2023, either in person or via an online conferencing system (Zoom, Zoom Video Communications, San Jose, CA), depending on participant preferences. Nine participants were interviewed individually, while two participants who worked in the same section of the municipality and shared similar experiences during the pandemic were interviewed in pairs. Pair interviews were chosen for these participants as mutual discussion was expected to elicit richer, co-constructed insights, grounded in shared experiences and complementary perspectives. Each interview lasted approximately 75 minutes with the following questions asked from a semi-structured interview guide: “How many years of experience do you have as a PHN?”; “How many years of experience do you have in providing maternal and child health welfare services during the COVID-19 pandemic?”; “Would you tell me in as much detail as possible about outreach activities you conducted to support specific pregnant women and families in need of assistance during the pandemic?"; "What challenges did you encounter in these outreach practices?"; and "What resources do you think are needed to address these challenges?". With the consent of the participants, the interviews were recorded or videotaped.

Data analysis

The recorded interview data were transcribed verbatim. Transcripts were carefully reviewed, and the content was coded with a focus on “the outreach implemented by PHNs for childcare support during the COVID-19 pandemic,” “challenges faced,” and “resources needed to address these challenges,” ensuring the meanings were preserved. The coding process was conducted by two researchers who independently analyzed the data to ensure a comprehensive interpretation of participants' experiences. Initially, each researcher became familiar with the data through multiple readings, and then open coding was used to identify key concepts and patterns. To increase the rigor of the analysis, an inter-coder reliability assessment was conducted. After the first round of coding, the researchers compared their codes to assess consistency. Disagreements were discussed in collaborative meetings until a consensus was reached to ensure a reliable interpretation of the data. Once coding was complete, codes were grouped into broader categories based on similarities and recurring patterns. This process involved several rounds of refining and collapsing codes into overarching categories that best represented the data.

For the pair interviews, a two-step analysis approach was employed to account for the unique dynamics of dyadic interaction. First, the transcripts were analyzed using the same analysis framework as individual interviews to identify themes at the participant level. Second, interaction patterns within the pair interviews, such as agreement, elaboration, or divergence between participants, were examined to capture insights unique to the pair context. This process allowed differentiation between themes arising from the interaction and those consistent with individual accounts.

To mitigate potential interaction effects, such as participants aligning their narratives or withholding information, the interview guide included prompts designed to encourage individual reflection within the pair setting. The researchers repeatedly reviewed the analysis process to ensure reliability and rigor.

Ethical considerations

Ethical approval for this study was obtained from the Ethics Review Committee of the researchers' affiliated university (Approval No.: 2022F25; Approval Date: March 16, 2023). The participants were informed both orally and in writing about anonymity and data-handling methods, and consent was obtained.

## Results

The attributes of the participants

The attributes of the participants are shown in Table 1. All participants were female. Based on the classification by Saeki et al. [12], the years of practical experience as PHNs were categorized as follows: three participants had less than six years of experience (novice phase), seven participants had six to 21 years of experience (mid-career phase), and one participant had >21 years of experience (veteran phase). Regarding involvement in maternal and child health welfare services during the COVID-19 pandemic, one participant had less than two years of experience, two participants had two to three years of experience, and eight participants had more than three years of experience. Regarding municipal population size, one participant was from a municipality with <50,000 residents, three from municipalities with 50,000-100,000 residents, five from municipalities with 100,000-500,000 residents, and two from municipalities with >500,000 residents.

**Table 1:** Attributes of the participants. PHNs: public health nurses; COVID-19: coronavirus disease 2019.

PHN	Experience years of PHN	Experience years involved in maternal and child health welfare services during the COVID-19 pandemic	Municipal population size	Group based on municipal population size
A	<6 years	2–3 years	>500,000	Large
B	<6 years	<2 years	100,000–499,999	Upper medium
C	<6 years	2–3 years	>500,000	Large
D	6–21 years	>3 years	100,000–499,999	Upper medium
E	6–21 years	>3 years	100,000–499,999	Upper medium
F	>21 years	>3 years	<50,000	Small
G	6–21 years	>3 years	100,000–499,999	Lower middle
H	6–21 years	>3 years	100,000–499,999	Lower middle
I	6–21 years	>3 years	50,000–99,999	Lower middle
J	6–21 years	>3 years	50,000–99,999	Lower middle
K	6–21 years	>3 years	50,000–99,999	Lower middle

The features of outreach practices performed by PHNs

The outreach practices conducted by PHNs to support childcare for specific pregnant women and families requiring assistance during the COVID-19 pandemic were categorized into 26 subcategories and five overarching categories (Table 2). The categories are listed below and explained using subcategories, followed by representative quotes from the PHNs.

**Table 2:** Categories and subcategories. PHNs: public health nurses; COVID-19: coronavirus disease 2019.

Categories	Subcategories
Category A: maintaining face-to-face support through home visits	Concerning the isolation of residents
	Recognizing the lack of postpartum care
	Recognizing increased needs for revisits by PHNs
	Assessing situations through face-to-face visits
	Proposing home visits if deemed necessary
	Pretending to visit for another reason to assess the situation
	Minimizing infection risks while conducting face-to-face visits
	Providing support tailored to the needs of the target households
	Addressing the needs of multicultural families through face-to-face visits
Category B: imperatively maintaining connections with clients through diverse communication channels, thereby securing and sustaining trust	Building relationships with clients via minimal contact
	Establishing connections with isolated families
	Identifying the needs of the clients
	Focusing on relationship-building in the preliminary stages before addressing core issues
	Selecting words and explanations that would not cause discomfort to the clients
	Fostering relationships through casual conversations
	Gradually deepening trust through written correspondence
	Building relationships with the children to gain acceptance from the parents
Category C: adapting to suit individual circumstances and responding effectively to emergent situations	Balancing infection control measures with effective support
	Providing flexible assistance tailored to the needs of the clients
	Adapting their engagement approaches to suit individual circumstances
	Responding flexibly during emergencies
Category D: utilizing and enhancing support networks within the community	Guiding clients to local resources to facilitate their use
	Leveraging collaboration with multiple agencies to enhance the quality of support
Category E: incorporating methodologies such as information and communication technology (ICT) and digital tools while innovating client-centered approaches	Utilizing online tools accepted by clients
	Adopting new methods such as online consultations
	Using email as an alternative to in-person visits

Category A: maintaining face-to-face support through home visits

PHNs emphasized assessing situations through face-to-face visits and maintaining the frequency and focus of such visits, even during the COVID-19 pandemic. They expressed concerns about the isolation of residents and the limited postpartum care available. Nurses recognized the need for continued face-to-face visits. Even when visits were declined, nurses employed creative strategies, such as proposing visits for other reasons, to assess family situations. Moreover, infection control measures were prioritized during visits.

Concerning the Isolation of Residents

PHN B: "I think isolation has become a significant issue, especially when looking at it from the perspective of pregnancy."

Recognizing the Lack of Postpartum Care

PHN H: "Since the onset of the COVID-19 pandemic, prenatal education at maternity hospitals - like parenting classes or newborn bathing practice - has drastically reduced. There were times when almost none of it was happening."

Recognizing Increased Needs for Revisits by PHNs

PHN H: "Home visits have increased. Or rather, follow-up visits - the second visits - have become more frequent. It feels like there are a lot more cases that cannot be resolved with just one visit."

Assessing Situations Through Face-to-Face Visits

PHN E: "Even before the COVID-19 pandemic, we provided personalized guidance and support tailored to the lives of specific pregnant women. It is not just about helping after the baby is born but figuring out what makes life easier for them."

Proposing Home Visits if Deemed Necessary

PHN C: "Our workplace did not really hold back on home visits. Even if families refused, we kept proposing a visit if we thought it was necessary."

Pretending to Visit for Another Reason to Assess the Situation

PHN A: "When visiting remote areas, I would check on families while also visiting daycare centers or children centers. It was a way to observe their situation without making it too obvious."

Minimizing Infection Risks While Conducting Face-to-Face Visits

PHN D: "Many pregnant women were concerned about COVID-19. We followed strict infection control measures during visits, shortened visit times, and ensured everything was wrapped up quickly."

Providing Support Tailored to the Needs of the Target Households

PHN F: "For families in need, especially specific pregnant women, we provided detailed nutritional guidance and other forms of support during home visits."

Addressing the Needs of Multicultural Families Through Face-to-Face Visits

PHN E: "Foreign residents seemed particularly scared of COVID-19. They often lacked access to clear, necessary information, so face-to-face communication was crucial."

Category B: imperatively maintaining connections with clients through diverse communication channels, thereby securing and sustaining trust

PHNs worked to build relationships with clients, even through minimal contact, and establish connections with isolated families. They prioritized identifying the needs of the clients and focused on relationship-building in the preliminary stages before addressing core issues. To achieve this, they carefully selected words and explanations that would not cause discomfort to the clients, fostered relationships through casual conversations, and gradually deepened trust through written correspondence. In addition, they employed family support techniques, such as building relationships with the children to gain acceptance from the parents.

Building Relationships With Clients via Minimal Contact

PHN A:"Even if they said, 'You do not need to come inside,' or kept the chain lock on, I always made sure to meet them somehow."

Establishing Connections With Isolated Families

PHN C: "There is one case I remember really well because it turned out great. The family was isolated and often labeled as a 'child abuse case.' It felt like every little issue was reported to child welfare services."

Identifying the Needs of the Clients

PHN J: "Honestly, there were times when we felt, ‘This situation seems dangerous; they must be struggling’; however, from their perspective, they might not have felt that way at all."

Focusing on Relationship-Building in the Preliminary Stages Before Addressing Core Issues

PHN K: "In the beginning, I focused on building trust and ensuring they would not reject contact in the future. Those early stages were crucial before diving into deeper issues."

Selecting Words and Explanations That Would Not Cause Discomfort to the Clients

PHN F: "I always try to use non-confrontational language and explain things in a way that is easy to understand."

Fostering Relationships Through Casual Conversations

PHN J: "Whenever I had the chance to meet them, I would engage in small talk and highlight things they were doing well."

Gradually Deepening Trust Through Written Correspondence

PHN A: "Over time, through exchanging letters, they started to open up and communicate more."

Building Relationships With the Children to Gain Acceptance From the Parents

PHN C: "Some families struggled to connect with their children. If they saw me as someone who could engage with their child, it made it easier for them to trust me."

Category C: adapting to suit individual circumstances and responding effectively to emergent situations

Despite contact restrictions that limited the scope of support, PHNs balanced infection control measures with effective assistance, tailoring their approaches to the needs of the clients. They adapted their engagement strategies to suit individual circumstances, responded flexibly to emergencies, and maintained connections through indirect methods, achieving tangible outcomes during the pandemic.

Balancing Infection Control Measures With Effective Support

PHN E: "Unless it was urgent, we held off on visits. However, for those who really needed it, we visited while following infection control measures."

Providing Flexible Assistance Tailored to the Needs of the Clients

PHN I: "I tried to identify their needs. If they were struggling financially, I guided them to available resources."

Adapting Their Engagement Approaches to Suit Individual Circumstances

PHN J: "The approach we took varied depending on the situation and the reports we received."

Responding Flexibly During Emergencies

PHN B: "Sometimes I would coincidentally meet families during daycare drop-offs or pick-ups, making it seem unplanned."

Category D: utilizing and enhancing support networks within the community

PHNs actively guided clients to local resources to facilitate their use and leveraged collaboration with multiple agencies to improve the quality of support, effectively utilizing community-based support networks.

Guiding Clients to Local Resources to Facilitate Their Use

PHN F: "Families who moved into the area often did not know about local resources. Explaining these made a big difference."

Leveraging Collaboration With Multiple Agencies to Enhance the Quality of Support

PHN B: "I gathered insights from daycare staff to share with mothers and prepared organized records for clear communication."

Category E: incorporating methodologies such as information and communication technology (ICT) and digital tools while innovating client-centered approaches

PHNs utilized ICT and digital tools effectively to maintain connections with clients. This included employing online tools accepted by clients, adopting new methods such as online consultations, and using email as an alternative to in-person visits, depending on available resources.

Utilizing Online Tools Accepted by Clients

PHN D: "We have also seen the introduction of services such as ‘Parenting Support Spaces’ with Zoom over the past 3 years. It has added another option for us to suggest as a place for consultations."

Adopting New Methods Such as Online Consultations

PHN H: "People seem to be quite receptive to using Zoom. I think it is because we usually proposed it to those who showed interest, so it has been well-received."

Using Email as an Alternative to In-Person Visits

PHN G: "I could only communicate with some people via email, and in the end, I could not visit their baby at home."

The challenges faced by PHNs

One prominent challenge faced by PHNs was the limitations associated with online support. Although the use of ICT and digital tools has advanced, some municipalities faced challenges in utilizing online tools owing to considerable barriers related to personal information protection. In addition, some clients were unwilling to accept online consultations, and PHNs expressed dissatisfaction with the depth and effectiveness of email communication with clients. The challenge of replicating the concreteness and depth of face-to-face support emerged as a key issue.

Considerable Barriers Related to Personal Information Protection

PHN H: "Handling personal information has been a significant challenge for us."

Challenges in Adopting Online Tools

PHN E: "We did not really use online tools. I think implementing online support proved challenging for us."

Clients Unwilling to Accept Online Consultations

PHN G: "I suggested Zoom consultations; however, as the babies grew older, they could not sit still during the sessions. Some clients also mentioned that preparing for Zoom sessions, like putting on makeup, felt burdensome."

Feeling a Lack of Depth and Satisfaction in Email Communication With Clients

PHN J: "Most interactions ended up being through email, and honestly, I often wondered, 'Is this really enough?'"

The resources and support systems required to address these challenges

PHNs expressed the need for integrating ICT as a complementary method to home visits. They noted that existing tools are limited to one-way communication from municipalities to clients. Nurses anticipated that enabling two-way online support between clients and PHNs could improve client engagement and responsiveness through digital tools. In addition, in an era where parenting records, such as feeding and nursing logs, are commonly managed via smartphone apps, nurses highlighted the necessity of a comprehensive support app that facilitates collaboration between families and PHNs. They called for the integrated use of childcare support apps to enhance the effectiveness of their support efforts.

Introduction of ICT

PHN G: "An official LINE account for our department and the city’s health center would be highly beneficial."

Use of ICT as a Complementary Method to Home Visits

PHN I: "Using Zoom or something similar saves the 1 h round trip, and it makes these meetings more efficient and effective. I think that is a real advantage."

Two-Way Online Support

PHN B: "For one-on-one sessions, I think many people feel nervous using Zoom. However, if we set it up more like a broadcast from our side - an online class that people can join using their smartphones and have the option to speak if they want - that might work better."

Improvement of Client Engagement and Responsiveness Through Digital Tools

PHN H: "Sometimes we send out surveys through an app; however, it would be great if people could respond to them quickly and easily."

Integrated Use of Childcare Support Apps

PHN D: "Over the past 2 or 3 years, it has become apparent that parenting records, like feeding logs, have shifted almost entirely to smartphones. I think it would be great if some comprehensive parenting app could cover all aspects of childcare."

## Discussion

The features of outreach practices performed by PHNs

The results of this study revealed key characteristics of the outreach practices conducted by PHNs during the COVID-19 pandemic.

First, the continuation of face-to-face home visits emerged as a key finding. This contrasts with prior studies [9] reporting a decline in home visits during the pandemic, challenging initial assumptions. For specific pregnant women and families requiring support, face-to-face interactions were critical for accurately assessing their needs and delivering tailored interventions. PHNs made efforts to sustain necessary visits while minimizing infection risks, which became the cornerstone of their outreach efforts. Online support proved insufficient for comprehensively assessing living conditions, such as observing home environments or surrounding areas [13], and visually evaluating situations [14,15]. Consequently, nurses prioritized direct, face-to-face support to meet the needs of isolated families who might lack the resources or confidence to seek help [16]. This continued support was particularly essential during the pandemic.

Second, building trust with clients was identified as a critical component across various healthcare settings, consistent with findings from international studies. Nurses employed methods such as fostering relationships through casual conversations and gradually building trust through written correspondence to maintain connections, even under restricted contact conditions. This aligns with studies from Italy and Australia, where nurses reported that maintaining personal connections through informal communication helped alleviate patient anxiety and fostered trust during the COVID-19 pandemic [17,18]. Mothers facing underlying challenges often struggle to accept support, making relationship-building essential [16]. Nurses in this study began by establishing connections with specific pregnant women, which laid the foundation for continuous support throughout pregnancy and parenting [19].

In addition, nurses leveraged established support networks to enhance the quality of assistance by collaborating with multiple organizations, including private entities. Flexible and innovative approaches helped mitigate pandemic-related constraints and alleviate the isolation of clients. Many parenting services, such as private childcare centers, were forced to close during the pandemic, leaving families more isolated. Despite these challenges, private organizations expanded their outreach through internet-based consultations and social media, increasing access to parenting support [20]. However, private organizations often faced regional limitations and struggled to assist high-risk households. This underscores the importance of utilizing support networks that bridge private organizations and public administrations to effectively serve high-risk families during crises.

Another notable feature was the use of online tools such as Zoom, LINE, and Instagram. As maternal and child health services adapted during the pandemic, some tasks transitioned online [20,21]. This enabled certain levels of support to reach remote areas and households where visits were challenging. While face-to-face support remains essential for comprehensive assessments and building trust, ICT tools can serve as a valuable complement by enabling follow-up interactions and providing timely information to clients in remote or restricted-access areas. Online tools allow nurses to provide evidence-based guidance and support while reducing the anxiety of clients through two-way communication [22]. The integration of ICT and online tools into outreach activities showed potential for broad dissemination of information and alleviation of parenting-related anxieties.

Challenges faced by PHNs

This study identified two major challenges: balancing infection control with face-to-face support and addressing the limitations of online support. Nurses' efforts to minimize infection risks during face-to-face visits and adapt support to restrictions imposed by contact limitations highlight these challenges. Continuing visits to high-risk households required navigating infection risks while maintaining trust with clients. This aligns with prior findings noting the difficulty of providing support when clients refused visits or interviews owing to fear of infection [23].

In addition, the limitations of online consultations were evident in cases where clients declined online support or where nurses felt that email exchanges lacked depth and effectiveness. Some parents preferred in-person consultations, citing the reassurance provided by direct interactions [24]. The challenge of replicating the concreteness and depth of face-to-face support with online tools further complicated outreach efforts. Addressing the needs of families without access to online environments emerged as a considerable challenge, consistent with findings on pandemic-related support for pregnant and postpartum women [9].

Needs for overcoming challenges

To overcome these challenges, PHNs emphasized the importance of integrating digital tools, adopting flexible communication methods, and balancing data protection with effective utilization. They highlighted the need for the integrated use of childcare support apps, addressing barriers posed by personal information protection. A centralized system for managing visit records, support plans, and progress tracking was deemed essential, along with communication tools adaptable to both online and offline contexts. Such systems would enable nurses to select the most effective support methods for each family. Strengthening data management systems to protect personal information while allowing efficient support is also critical. These initiatives align with studies on advancing public health through digital technology [25].

Limitations and future directions

This study analyzed narratives from PHNs with diverse practice experiences and municipalities of varying population sizes. However, the findings may not fully reflect nationwide conditions. First, we need to discuss how regional differences in healthcare practices and sample size may have influenced the findings. Specifically, differences in healthcare infrastructure, local policies, and cultural practices between regions may have influenced PHN behaviors and experiences during the pandemic. These factors may limit the generalizability of our findings. Second, the lack of comparison with outreach practices of other professions is a limitation. Third, while the combination of in-person and online interviews provided flexibility, the use of virtual interviews introduced certain limitations that should be acknowledged, such as technical issues and the inability to fully observe nonverbal cues. Despite these challenges, efforts were made to foster a comfortable and confidential atmosphere for all participants, regardless of the interview mode. In addition, offering both in-person and virtual options allowed participants to choose the format they were most comfortable with, which helped ensure diverse participation and enriched the overall data.

To address these limitations, we recommend that future studies include larger and more diverse participants from different countries and regions. Including PHNs from both urban and rural settings and from different healthcare systems will provide a broader understanding of the challenges and strategies in different contexts. In addition, comparative studies between regions with different healthcare infrastructures could highlight how systemic factors influence the reach of PHNs. In the long term, the results of this study can inform PHN training programs by emphasizing the importance of adaptability, the use of digital tools, and strategies for building trust in crisis situations. The findings may also guide health policy development by highlighting the need for flexible support systems that can function effectively during public health emergencies. In addition, the findings could help strengthen public health infrastructure in rural and underserved areas to ensure that outreach strategies are resilient and effective during pandemics and other crises.

## Conclusions

The key characteristics of the outreach practices conducted by PHNs to support specific pregnant women and families requiring assistance during the COVID-19 pandemic included maintaining face-to-face support through home visits, building trust with clients, overcoming pandemic-related restrictions and limitations through flexible support and adaptations, utilizing and promoting support networks, and leveraging ICT and digital tools. Concurrently, challenges such as balancing infection control with face-to-face support and the limitations of online support were highlighted.

To address these challenges, PHNs emphasized the importance of integrating digital tools, adopting flexible communication methods, and balancing personal data protection with effective data utilization. These findings offer valuable insights for supporting specific pregnant women and families requiring assistance in future pandemics or disasters.
